# Arrhythmias in COVID-19

**DOI:** 10.14797/mdcvj.1039

**Published:** 2021-12-15

**Authors:** Summit Pandat, Zhihao Zhu, Stephanie Fuentes-Rojas, Paul Schurmann

**Affiliations:** 1 Houston Methodist DeBakey Heart & Vascular Center, Houston Methodist Hospital, Houston, Texas, US; 2 Houston Methodist Hospital, Houston, Texas, US

**Keywords:** arrhythmia, COVID-19, coronavirus, atrial fibrillation

## Abstract

The coronavirus pandemic remains a major public health burden with multisystem disease manifestations. There has been an ongoing global effort to better understand the unique cardiovascular manifestations of this disease and its associated arrhythmias. In this review, we summarize the current data on incidence and outcomes of arrhythmias in the acute and convalescent period, possible pathophysiologic mechanisms, and medical management. Sinus bradycardia—reported in multiple observational studies in the acute infectious period—stands out as an unexpected inflammatory response. Atrial fibrillation has been noted as the most common pathologic arrhythmia and has been shown to be a poor prognostic marker in multiple cohorts. In the convalescent period, long-term complications such as postural orthostatic tachycardia syndrome and inappropriate sinus tachycardia have been described.

## Introduction

Coronavirus disease 2019 (COVID-19) has had a profound impact on global health for the last 2 years. Medical providers have faced numerous challenges in understanding the pathophysiology, complications, and management of the disease and in providing patient care while minimizing exposure. Cardiac manifestations of this disease have taken on many forms, including myocardial infarction, viral myocarditis, and arrhythmias.^1^ COVID-19–related arrhythmias were initially described in data from Wuhan, China, published in February 2020. In their study population of 138 hospitalized COVID-19 patients, arrhythmias occurred at a rate of 16.7% in the overall population and 44.4% in those in the intensive care unit.^2^ Since that initial data, multiple centers have sought to better clarify the types of arrhythmias associated with COVID-19 as well as their prognostic implications.

In this review, we aim to focus on the incidence, pathophysiology, and management of cardiac arrhythmias with COVID-19 infection in the acute and convalescent period.

## Arrhythmia in Acute COVID-19 Infection

### Pathophysiology of COVID-19

COVID-19 infection has been hypothesized to progress over multiple stages: an initial viral phase with viral constitutional symptoms, a secondary infection stage with viral entry into cells and replication primarily in type II pneumocytes leading to respiratory failure and acute respiratory distress syndrome (ARDS), and sometimes a more severe stage of multisystem failure with inflammation due to a cytokine storm.^3,4^ The inflammatory cytokines are known to cause arrhythmias due to sympathetic overactivation.^5^ Multiple cytokines including interleukin-6 (IL-6) and tumor necrosis factor-α can directly modify expression and function of cardiac potassium and calcium channels affecting the myocyte action potential, specifically in genetically predisposed individuals.^6^

Acute respiratory failure and ARDS following pulmonary infiltration from COVID-19 leads to hypoxia. The association between acute hypoxia and cardiac arrhythmias is well known and has been studied prior to the COVID-19 pandemic.^7^ Hypoxia can modify the function of L-type calcium channels affecting the plateau phase of action potential.^8^ Additionally, hypoxia eventually leads to anaerobic metabolism, which reduces intracellular pH and affects action potential duration.^9^ This has been shown to cause cardiac conduction tissue remodeling and anisotropy due to the effects on cardiac gap junction proteins connexin 40 and connexin 43, resulting in atrial and ventricular arrhythmia.^10,11^

Direct myocardial injury has been noted in a significant number of patients with COVID-19. Markers for cardiac injury and inflammation—including NT-proBNP, cardiac troponin I, and high-sensitivity C-reactive protein—have been significantly associated with severity of disease and indicate its potential for myocarditis.^2,12^ Acute viral myocarditis can cause direct cytotoxic remodeling of cardiac conduction tissue, microvascular ischemia, gap junction dysfunction, and ion-channel dysregulation, all increasing the risk for arrhythmias.^13^ Post-inflammatory myocardial fibrosis and scar have also been observed, which can increase the risk for re-entrant arrhythmias.^14^

Electrolyte abnormalities and fluid imbalance related to acute kidney injury and gastrointestinal symptoms have also been noted in a significant number of patients hospitalized for COVID-19.^2,12^ Significant derangements in potassium, magnesium, calcium, and phosphorus can all increase risk for new arrhythmia and exacerbate preexisting conduction disease. Additionally, early COVID-19 therapies such as hydroxychloroquine and azithromycin were known to carry an increased risk for arrhythmias.^15^ These medications can inhibit hERG potassium channels, which are responsible for the rapid delayed rectifier current (I_Kr_).^16^ The resultant prolongation of the cardiac action potential and increased early afterdepolarization leads to a prolonged QT interval and increased risk for torsade de pointes (TdP).^17^

Inflammation and hypoxemia are common mechanisms of cardiac arrhythmia but can be exacerbated by drugs or electrolyte imbalances that prolong QT. Some of these patients developed sinus bradycardia, which also plays a role in prolonged QT. This bradycardia is unexpected and different from the sinus tachycardia common in other patients with respiratory failure and hypoxemia. Although the exact mechanism of the sinus bradycardia is unclear, the pacemaker cell dysfunction at the sinus node is evident.^18^

**Figure 1:**
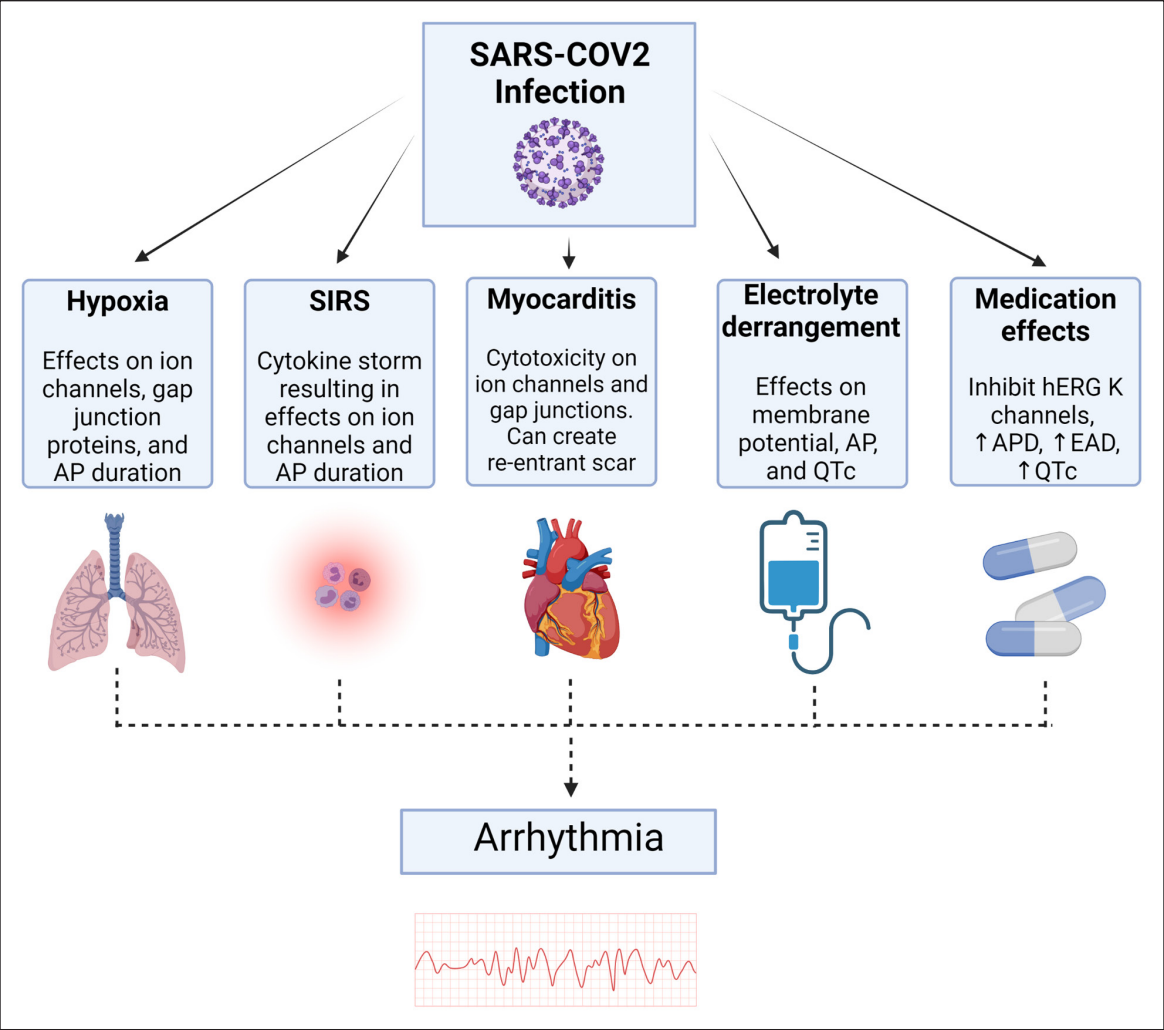
COVID-19 contributors to arrhythmogenesis. Created with *BioRender.com*. AP: action potential; SIRS: systemic inflammatory response syndrome; QTc: corrected QT interval; hERGk: human ether-a-go-go related gene; APD: action potential duration; EAD: early afterdepolarization

The sum of these various metabolic changes affects an individual’s risk for arrhythmia during the acute infectious period (***Figure 1***).

## Arrhythmogenesis of Medical Therapy in Acute COVID-19 Infection

Throughout the pandemic, a variety of therapies have been evaluated for their antiviral and/or anti-inflammatory effects or their efficacy in improving ARDS. However, many of these medications have been associated with an increased risk of arrhythmias. For example, the combination of hydroxychloroquine and azithromycin was shown to have an antiviral effect in a small study from France, and many providers used this early during the pandemic.^19^ Although it was granted emergency use authorization by the US Food and Drug Administration (FDA), this therapy was later revoked after larger randomized studies demonstrated no significant effect on mortality or clinical status for patients with COVID-19.^20^

The main concern regarding arrhythmia is the QT prolongation effect and increased risk for TdP when taking hydroxychloroquine/azithromycin. A few cohort studies showed relatively little risk, with only small-to-moderate increases to QT interval after a short course of treatment with hydroxychloroquine +/- azithromycin.^21,22^ In a systematic review by Jankelson et al. of 1,515 COVID-19 patients treated with hydroxychloroquine or chloroquine with and without azithromycin, about 10% of patients developed clinically significant QT prolongation (QTc > 500 ms or increase > 60 ms).^23^ They also found two documented cases of ventricular arrhythmia in patients with severe COVID-19 treated with high-dose chloroquine.^23^ Due to this risk, hydroxychloroquine and azithromycin are no longer recommended in the routine treatment of COVID-19 patients.

Combination lopinavir-ritonavir was also used early on as an experimental treatment for COVID-19 due to some evidence of efficacy during the previous outbreaks of SARS-CoV in 2003 and MERS-CoV in 2012.^24,25^ However, previous use in patients with the human immunodeficiency virus demonstrated significant bradyarrhythmia as well as tachy-brady syndrome.^26^ In a cohort of 41 COVID-19 patients with critical illness treated in ICUs, 22% developed significant bradycardia with one case of third-degree atrioventricular block. These bradyarrhythmia episodes were attributed to the therapy since they started within 48 hours of initiation and resolved after discontinuation, with no cases among patients who did not receive the therapy.^27^ Lopinavir-ritonavir is no longer recommended for COVID-19 after further studies failed to show significant benefit to mortality or clinical status.^28^

Remdesivir has now become the primary antiviral therapy used for COVID-19 after demonstrating modest benefit in improving clinical course and shortening time to recovery in moderate to severe cases.^29,30^ However, multiple case reports of bradyarrhythmia resulting from remdesivir treatment have been reported.^31,32,33^ In a review of the World Health Organization Case Safety Reports database, Toufchia et al. compared the incidence of bradycardia among COVID-19 patients treated with remdesivir versus those treated with hydroxychloroquine, lopinavir-ritonavir, tocilizumab, or glucocorticoids and found a significantly increased risk of severe bradycardia and of reporting bradycardia (OR 1.65; 95% CI, 1.23-2.22) in those treated with remdesivir.^34^

There are many other additional adjunct therapies being used in the management of COVID-19 patients. Tocilizumab, a potent IL-6 receptor antagonist, has had mixed results in showing benefit to hospitalized patients but is commonly used for its anti-inflammatory effects.^35,36^ Prior studies have shown an actual arrhythmic benefit with this medication because it tends to shorten the QTc interval, indicating that it may have a protective effect when other QTc-prolonging medications are being used.^9,37^ High-dose corticosteroids are also frequently used given their demonstrated mortality benefit in critically ill COVID-19 patients and lower relative cost.^38^ Generally, corticosteroids don’t confer an increased risk of malignant arrhythmias, but they were previously shown to increase the risk for the development of atrial fibrillation.^39^ For critically ill COVID-19 patients with ARDS, heavy sedation is a mainstay of therapy and involves use of paralytics and sedatives such as propofol and dexmedetomidine. Propofol, which is a known cardiac depressant, carries both anti- and proarrhythmic properties due to its ability to suppress both sympathetic and parasympathetic tone while also preserving gap junctions in ischemia.^40^ Dexmedetomidine, a selective alpha-2 receptor agonist, is commonly used in patients with high sedation requirements; it carries a known risk of bradycardia but may have some degree of favorable QTc shortening.^41,42,43^

## Incidence of Cardiac Arrhythmias in Acute Infection of COVID-19

Multiple centers have been actively engaged in efforts to better clarify the burden of arrhythmias associated with COVID-19. The ACOVID (Arrhythmias in hospitalized patients with COVID-19) study in Denmark recruited individuals over age 18 admitted with COVID-19 to one of six nearby hospitals in Copenhagen and prospectively monitored them for various arrhythmias. Out of only 54 patients, they found 9 events of cardiac arrest (one with TdP), 25 incident atrial fibrillation (AF) events, 9 bradyarrhythmias, and 10 nonsustained ventricular tachycardia (NSVT) events.^44^ In California, Cho et al. reviewed the telemetry data of 143 COVID-19 patients at Cedars-Sinai hospital and found that 39.9% had sinus tachycardia. Other common arrhythmias included premature ventricular contractions (PVCs) at 28.7%, NSVT at 15.4%, AF at 11.9%, and supraventricular tachycardia (SVT) at 5.6%. Malignant arrhythmias such as ventricular tachycardia/ventricular fibrillation (VT/VF) and atrioventricular (AV) block were rare, occurring in 0.7% to 1.4%. The only significant difference they found between survivors and nonsurvivors was a higher frequency (58.3%) of sinus tachycardia in nonsurvivors.^45^

A larger data set from Bhatla et al. at the University of Pennsylvania retrospectively evaluated 700 patients admitted between March 2020 and May 2020 and found arrhythmic events to be quite rare. They found nine cardiac arrests, six of which were pulseless electrical activity (PEA), one asystole, and one TdP. There were 25 incident AF events, 9 significant bradyarrhythmias, and 10 events of NSVT. There were no noted cases of AV block or sustained VT/VF. ICU status was found to be independently associated with incident AF in multivariate analysis (OR 1.05; 95% CI, 1.02-1.09). Heart failure was associated with bradyarrhythmias (OR 9.75; 95% CI, 1.95-48.65).^46^ ***Table 1*** summarizes incidence data and various management strategies.

**Table 1:** Arrhythmias in COVID-19. Incidence data based on available prospective and retrospective cohort data cited in text. AV: atrioventricular; AF/AFL: atrial fibrillation/atrial flutter; CCB: calcium channel blocker; BB: beta blocker; SVT: supraventricular tachycardia; AVN: atrioventricular node; PVCs: premature ventricular contractions; AADs: antiarrhythmic drugs; NSVT: nonsustained ventricular tachycardia; TdP: torsade de pointes; POTS: postural orthostatic tachycardia syndrome; ICD: implantable cardioverter defibrillator; IST: inappropriate sinus tachycardia


TYPE	REPORTED INCIDENCE (%)	COMMENTS	MANAGEMENT STRATEGIES

Sinus tachycardia	40-55%	Most common, appropriate in acute setting	COVID-19–directed treatment

Sinus bradycardia	5-25%	Likely a poor prognostic marker	Avoid AV nodal blockade Avoid dexmedetomidine if possible Temporary or permanent pacing if profound and unstable

AF/AFL	2-12%	Most common pathologic arrhythmia, poor prognostic marker	Rate/rhythm control strategies CCBs preferred over BBs to minimize bronchospasm

SVT	0.6-6%		Usual care with adenosine, AVN blockers, and cardioversion if unstable

PVCs	0-28%		No evidence for prophylactic AADs

NSVT	0-15%		No evidence for prophylactic AADs

Sustained VT/VF or TdP	0-1.4%	Usually only in critical illness	Defibrillation and AADs VT catheter ablation if AADs not tolerated ICDs for secondary prevention though unclear long-term benefit

AV block	0-1.4%	Usually only in critical illness, unclear if reversible	Temporary or permanent pacing

POTS	4-22%	Reported in the convalescent period due to dysautonomia	Nonpharmacologic: compression stockings, salt intake, exercise Pharmacologic: mineralocorticoids, alpha agonists, BBs, ivabradine

IST	3-4%	Reported in the convalescent period due to dysautonomia	BBs, ivabradine


### Atrial Arrhythmias

In a review of large cohort data, atrial arrhythmias stand out as the most common arrhythmia during the acute phase of COVID-19. A recent retrospective study of 3,970 COVID admissions at Mount Sinai Hospital found that atrial fibrillation/atrial aflutter (AF/AFL) occurred in 10% of patients overall and in 4% of those with no prior history of atrial arrhythmias. Furthermore, the occurrence of AF/AFL was associated with an increased mortality of 46% versus 26% of patients with no arrhythmias (*P* < .01).^47^ In multiple other studies, the occurrence of AF/AFL has similarly been noted to be a poor prognostic marker. For example, Ip et al. found that the occurrence of atrial fibrillation was an independent predictor of mortality with an OR of 4.8, *P* = .004.^48^ Mountantonakis et al. similarly found that the occurrence of AF carried a higher mortality of 54.3% versus 37.2%, *P* < .001. Within the AF group, mortality was also higher in those with new-onset AF versus those with a known history of AF (55.2% vs. 46.8%, *P* = .009).^49^

In cohort data, SVTs have rarely been reported. Yarmohammadi et al. noted that atrial arrhythmias occurred in 8% of 1,029 patients admitted with COVID-19, and within that group, 8% had SVTs, 5 cases had long RP tachycardia, and 2 had short RP tachycardia. The cases of long RP tachycardia were thought to most likely represent focal atrial tachycardia.^50^

### Ventricular Arrhythmias

Ventricular arrhythmias including VT/VF, NSVT, and PVCs have been reported to occur quite rarely in most study populations, although Cho et al. found PVCs and NSVT to be quite common within their cohort.^45^ Abrams et al. from Columbia University published a case series of 5 critically ill COVID patients who had VT/VF, three of which were refractory to amiodarone and defibrillation. Although these patients had risk factors for cardiovascular disease such as obesity, hypertension, and diabetes, none had any history of heart failure, left ventricular dysfunction, or evidence of infarct on electrocardiogram. The authors theorized that these VT/VF arrests were more likely secondary to iatrogenic factors such as permissive hypercapnia, electrolyte imbalances, and COVID-19–directed therapies.^51^ Shao et al. reviewed cardiac arrest data for COVID infections in Wuhan between January and February 2020 and found that 5.9% of cardiac arrests were due to a shockable rhythm such as VT/VF.^52^

### Bradyarrhythmias

Bradyarrhythmias such as sinus bradycardia associated with COVID-19 have been well described, yet cases of individuals needing temporary or permanent pacemaker implantation are rare.^53^ Kumar et al. looked specifically at the occurrence of absolute bradycardia (HR < 60) and profound bradycardia (HR < 50) associated with 1,053 admitted COVID-19 patients. Excluding patients on AV nodal blockers or with end of life bradycardia, the authors found that absolute bradycardia occurred in 24.9% of COVID-19 admissions and carried a mortality rate of 17.7%. Profound bradycardia occurred in 13% of patients and carried a mortality rate of 25.5%. They reported an odds ratio of 6.59 (95% CI, 2.83-15.36) for mortality in those with absolute bradycardia compared to individuals with a normal HR response.^54^

Complete AV block, though quite rare, was reported by Li et al. to occur at a rate of 1.5% in their cohort of patients from China. They found that any form of conduction block occurred at an overall rate of 11.9%, with first-degree AVB block and right bundle branch block being the most common, occurring at a rate of 3.7%.^55^

## Management of Cardiac Arrhythmia during COVID Infection

The medical management of arrhythmias during the acute COVID-19 infectious period has not deviated much from standard practice. For atrial arrhythmias, rate and rhythm control strategies have remained the mainstay of treatment. However, the use of beta blockers for rate control has been a common concern due to the potential for bronchospasm in the active phase of COVID-19 infection; thus, many have used nondihydropyridine calcium channel blockers such as diltiazem as an alternative to avoid this potential side effect.^46^ Rhythm control with amiodarone is still quite common, reported by Berman et al. as 29% of COVID-19 patients referred for electrophysiology consultation at Columbia University.^56^

For ventricular arrhythmias, specific QTc and electrolyte monitoring is required during the acute phase of COVID-19 infection, especially if proarrhythmogenic drugs are used. The incidence of ventricular arrhythmia has primarily been associated with critically ill patients in the setting of ventilation, permissive hypercapnia, and electrolyte imbalances. Thus, efforts would best be focused towards minimizing the impact of these factors. There have been some reported cases of VT storm where substrate-based VT catheter ablation was efficacious. This may prove to be a viable alternative in those who may not be suited for antiarrhythmics.^57^

In bradyarrhythmias, permissive hyperthermia to avoid hypothermia can be useful in minimizing sinus bradycardia. The use of inotropic agents such as dopamine or dobutamine can additionally be used for supportive care. Also, temporary pacemakers have been used when all other measures fail.^53^ It is not clear whether the occurrence of profound bradycardia and high-degree AV block is potentially reversible when a patient recovers from their acute infection.

## Arrhythmia in the COVID-19 Convalescent Period

There is an ongoing effort to better understand post COVID-19 condition—that is, the long-term effects of acute infection in COVID-19 patients. Many arrhythmias have been anecdotally noted in this period, including ventricular arrhythmias such as PVCs and ventricular tachycardia, although large data sets on this are still quite limited.

Dysautonomia in particular has been documented quite frequently in the post-infectious period and carries its own long-term management implications. The ALBACOVID registry in Spain retrospectively obtained neurologic data from COVID-19 admissions and found that dysautonomia occurred at a rate of 2.5%.^58^ Shouman et al. evaluated patients with COVID-19 infection who had been referred for autonomic testing at the Mayo Clinic and found that 22% of them fulfilled criteria for postural orthostatic tachycardia syndrome (POTS). Only one patient in their review was diagnosed with inappropriate sinus tachycardia (IST).^59^ In a cohort of 200 patients with symptoms persisting 3 months after COVID-19 infection, Llach et al. found that IST or POTS criteria were met in 17% of patients, with 26 cases of IST and 8 cases of POTS. These patients had no structural heart disease identified on echocardiography but had significantly diminished exercise capacity on 6-minute walk test and altered heart rate variability on 24-hour Holter monitoring.^60^

Mechanistically, it is unclear why dysautonomia persists into the convalescent period of COVID-19 infection; however, similar to other viral syndromes, the cause is theorized to be a post-infectious autoimmune phenomenon. As with other patients with dysautonomias, these conditions can be managed with both pharmacologic and nonpharmacologic measures. Nonpharmacologic measures, particularly for POTS, include compression stockings, increased salt intake, and regular exercise to improve physical conditioning. Pharmacologic measures for POTS—depending on the primary etiology—can include mineralocorticoids, alpha agonists, beta blockers, or ivabradine. Similarly, IST can be managed with either beta blockers or ivabradine, although ivabradine use is off label for both IST and POTS and not FDA approved for these conditions.^57^

## Conclusions

COVID-19 continues to have a global impact both during and after infection. As our understanding of this disease evolves, we will hopefully have better insights into its unique pathophysiology and arrhythmic complications. Atrial fibrillation has been documented in multiple studies as the most common pathologic arrhythmia in COVID-19 infection and has been shown to be a poor prognostic marker, associated with increased risk for mortality especially in new-onset cases. Bradyarrhythmia also tends to be a common arrhythmia in the acute phase and also is associated with poor prognosis. As we move into the post-COVID period, more data is needed on the long-term arrhythmic effects of the disease to gain a better understanding of how to manage patients in the convalescent period. Additionally, further studies will be needed to monitor for long-term arrhythmias associated with new therapies.

## Key Points

- The most common arrhythmias in the acute infectious period of COVID-19 are sinus tachycardia (nonpathologic) and atrial fibrillation (pathologic).
- Atrial fibrillation is a poor prognostic marker and has been associated with higher mortality, especially in new-onset cases.
- Malignant arrhythmias such as ventricular tachycardia, ventricular fibrillation, and atrioventricular block are rare.
- Special interdisciplinary care is needed to balance the medical management of these patients to minimize iatrogenic contributions to arrhythmias.
- Inappropriate sinus tachycardia and postural orthostatic tachycardia syndrome seem to be common in the convalescent period of COVID-19 infection as a result of post-infectious dysautonomia.
